# Qualitative Feasibility and Usability Study of an Ecological Momentary Assessment (EMA) Smartphone Application in Binge Eating Disorder Treatment

**DOI:** 10.3390/nu18183046

**Published:** 2026-09-17

**Authors:** Pauline W. Jansen, Ivonne P. M. Derks, Judith van Russen Groen, Jeffrey van der Starre, Joran Jongerling, Hans W. Hoek, Machteld Wery, Femke Truijens

**Affiliations:** 1 Department of Psychology, Education, and Child Studies, Erasmus University Rotterdam, Burgemeester Oudlaan 50, 3062 PA Rotterdam, The Netherlands; i.derks@ucl.ac.uk (I.P.M.D.); j.vanrussengroen@zorgspectrum.nl (J.v.R.G.); truijens@essb.eur.nl (F.T.); 2 Department of Child and Adolescent Psychiatry/Psychology, Erasmus MC University Medical Center, Wytemaweg 8, 3015 CN Rotterdam, The Netherlands; 3 Research Department of Behavioural Sciences and Health, University College London, 1-19 Torrington Place, London WC1E 7HB, UK; 4 Parnassia Psychiatric Institute, Max Euwelaan 70, 3062 MB Rotterdam, The Netherlands; j.vanderstarre@psyq.nl (J.v.d.S.); w.hoek@parnassiagroep.nl (H.W.H.); wery@emergis.nl (M.W.); 5 Tilburg School of Social and Behavioral Sciences, Tilburg University, Professor Cobbenhagenlaan, 5037 DB Tilburg, The Netherlands; j.jongerling@tilburguniversity.edu; 6 Department of Psychiatry, University Medical Center Groningen, University of Groningen, Hanzeplein 1, 9713 GZ Groningen, The Netherlands; 7 Department of Epidemiology, Mailman School of Public Health, Columbia University, 722 W 168th St, New York, NY 10032, USA; 8 Faculty of Psychology and Educational Sciences, Ghent University, Henri Dunantlaan 2, 9000 Ghent, Belgium

**Keywords:** binge eating disorder, therapy, m-health, EMA, feasibility

## Abstract

Objective: Cognitive behaviour therapy (CBT) for binge eating disorder (BED) is fairly effective, yet relapse remains problematic. Treatment outcomes may be improved if patients gain more insight into symptom patterns. In this qualitative study embedded in BED treatment, we evaluated user experiences with an Ecological Momentary Assessment (EMA) smartphone application to self-monitor affect and binge eating episodes in daily life. Methods: Participants were 20 adults who received group-CBT for BED at a mental health institute in the Netherlands. Via an EMA smartphone app, the participants filled out 5 micro-questionnaires daily for 6 weeks. An overview of mood fluctuations and binge eating episodes was graphically displayed in the app and discussed with the therapist. Semi-structured interviews were conducted with 16 participants and 5 practitioners to evaluate feasibility and usefulness. Results: Qualitative analyses of the interviews showed that the app was perceived as user-friendly, yet invasive due to many registrations. This was reflected in low response rates (mean: 31.1%), with only 9 participants reaching a response rate above 30% (>65 of 210 EMA micro-questionnaires completed). Some participants thought the daily registrations were insightful, but some also perceived the registrations as confronting and shameful, suggesting potential iatrogenic effects. Conclusions: This study offers a real-world example of using EMA in treatment and provides meaningful insights for developers of behaviour change-focused EMA tools. The experiences of the participants and practitioners were mixed, yet several indicated improved reflection and awareness of (fluctuations in) emotions. Given the cautiously positive feedback from several participants and therapists, we recommend a carefully designed evaluation, including a shorter assessment period, a control group and an assessment of emotional awareness.

## 1. Introduction

Binge eating disorder (BED) is the most common eating disorder, with detrimental effects on individuals’ health and well-being, as it may lead to obesity, depressive symptoms and substance abuse [1]. Treatment of BED mostly consists of elements of cognitive behaviour therapy (CBT) [2]. Group-based or individual CBT is effective in alleviating BED symptoms, with abstinence rates after one year varying between 46 and 52% for psychotherapy [2]. However, several individuals only reach partial remission and may face relapses in the longer term. This may be due to a host of different factors, including underlying alexithymia and problems with emotion regulation [3,4]; thus, treatments that more effectively target the maintenance mechanisms of BED are needed [5,6].

Beyond other aetiological factors, mood changes and emotion regulation processes seem to play a key role in binge eating among most individuals with BED [3,7,8,9]. The affect regulation model of binge eating posits that binge eating plays a role in the regulation of one’s negative affect, suggesting that binge eating episodes are preceded by a deterioration in affect or stress, while mood tends to improve after such an episode [7]. Many studies have tested this model by utilizing Ecological Momentary Assessments (EMA) to capture momentary ratings and repeated assessments of emotions and activities in daily life. A recent comprehensive meta-analysis integrated results from 59 EMA studies, generally supporting the notion that deteriorations in emotions precede and thus seem to trigger binge eating [9]. Yet, evidence for the emotional consequences of binge eating depended on the type of analyses: the level of negative affect was generally worse at the first post-binge rating, but temporal trajectories—based on multilevel modelling in more recent studies—suggest slight improvements after binge eating [9]. Although these findings highlight the role of (fluctuations in) emotions, only a few studies examined the clinical utility of EMA in BED treatment.

Le Grange et al. (2002) conducted a pilot study in which CBT treatment with and without EMA was compared among 41 individuals with BED [10]. EMA was implemented in the first two weeks of treatment using a wristwatch that beeped several times per day and a pre-printed diary. After each week, the participants discussed the pattern of their binge eating episodes with the therapist. In 77% of these individuals (compared to 47% of the control group), the binge eating frequency reduced by half, but findings were not statistically significant [10]. Another pilot study among 16 individuals with BED or Bulimia Nervosa used an EMA smartphone application as an add-on during emotion-focused CBT [11]. The app registered daily emotions as cues for disordered eating and, alongside this, delivered customized coping strategies during moments of risk. The participants found the app acceptable as treatment augmentation and indicated that it helped accomplish therapy goals, yet paradoxically, compliance (daily registrations) was too low to trigger intervention strategies [11]. A few other studies investigated the use of ecological momentary interventions as an add-on to CBT treatment, encompassing direct delivery of therapeutic support via smartphone applications, sometimes based on self-initiated self-monitoring [12,13]. For these studies, it is, however, difficult to determine the role of EMA itself.

Taken together, EMA is a promising augmentation to current eating disorder therapies because the monitoring of affect and binge eating may provide insight into individual symptom patterns that can be used to tailor treatment [14]. Yet, as initial evidence suggests possible issues with compliance, it is necessary to value individuals’ and therapists’ experiences to define feasibility and acceptability [11] before EMA is incorporated as a tool during treatment. The current qualitative study evaluated the experiences of participants and therapists with EMA as an augmentation tool for BED treatment.

## 2. Materials and Methods

### 2.1. Design and Participants

This study was conducted at the Eating Disorder outpatient clinic of the Parnassia Groep, a mental health institute in the Netherlands. All individuals who enrolled in CBT for BED between 1 August 2021 and 1 August 2022 (*n* = 35, five treatment groups) were asked to use the EMA smartphone application as part of their treatment. This treatment is indicated for individuals with a confirmed DSM-5 BED diagnosis. Exclusion criteria for the treatment—and thus for the current study—were insufficient Dutch language skills, medication use that is known to trigger binge eating (e.g., corticosteroids or antipsychotics) and a suspected monogenetic pathology as the cause of binge eating. Twenty individuals consented for their EMA data to be analysed in the study, of whom 16 also consented to participate in a qualitative, semi-structured interview about their experiences with the app. All participants were still following the treatment when the interviews took place. Of the 8 therapists and treatment coordinators (referred to as “practitioners”), 5 consented to participate in an interview.

### 2.2. Procedure

Participants enrolled in treatment as usual, a group-based CBT for BED consisting of 20 (bi-)weekly sessions [15]. In the first session, an involved researcher from the Parnassia Groep introduced the EMA app and the study, and handed out patient information and an informed consent form. The participants could consent to the use of their EMA data in our study. Additionally, we asked individuals and practitioners for their consent to participate in an interview on the usability of the app. In the second session, consent forms were collected. Then, all individuals were asked to download the app as it was part of the treatment protocol for everyone, but we only used data from those consenting to participate in the study. The participants received personal login codes and instructions to download the app. During weeks 2–7, the participants received daily invitations to fill out micro-questionnaires on emotions and binge eating in daily life. The reports of each participant were automatically included in a personal graph in their app to offer insight into their patterns of emotions and binges. An example of a fictitious personal graph is shown in the Supplementary Materials (Figure S1).

The registration data was not accessible to the practitioners, but during the weekly therapy sessions, the participants could discuss the registration procedure and the graph with the practitioners. The primary purpose of the app was to increase awareness of (patterns in) emotions and behaviours, by helping individuals monitor these in daily life. While the app was not designed to modify the protocolized CBT intervention elements, the EMA-derived information could be leveraged by the practitioners to enrich therapeutic discussions, for example, by reminding patients of specific emotional experiences or recurring behavioural patterns. After week 7, interviews with the participants and practitioners took place. The semi-structured one-on-one interviews on user experiences (feasibility, usefulness and added value) lasted 30–45 min, were conducted by a separate interview team consisting of master’s students in Clinical Psychology and were audiotaped and transcribed by them afterwards. Interviewers were trained by a senior qualitative researcher, which included, for example, reflexivity training and cross-check interview questioning. Because the interviews focused on an intervention developed by the research team, we took several steps to further enhance reflexivity and minimize potential bias. Therapists, interviewers, and researchers each conducted part of the project, but worked independently from each other. Moreover, although demographically the team was quite homogeneous (different sexes, yet all White and highly educated), the clinical and academic background reflected more diversity (educational background; professional role, i.e., clinicians and academics; expertise in binge eating; and career stage). Data analysis was primarily conducted by non-clinically practising researchers and discussed with clinical team members to incorporate multiple perspectives and to ensure a neutral and nuanced perspective on themes and coding.

### 2.3. Measures

#### 2.3.1. General Characteristics

Information on sex and age was retrieved from patient records. At intake, the highest obtained educational level was recorded, and height and weight were measured. Symptom severity pre- and post-treatment was self-reported on the validated Eating Disorder Examination Questionnaire (EDE-Q) [16,17].

#### 2.3.2. EMA Measures

A smartphone application for EMA was developed by the research team in cooperation with the tech company Innovattic. Both developers and researchers tested the app multiple times before deploying it in the intervention. Security requirements were tested (including a penetration test by an external company) and approved by the security officer of Erasmus University Rotterdam. The app, called Daily Diary, was installed by the participants on their smartphones and prompted the participants, via text message or notification, for data entry for six consecutive weeks at five semi-random intervals each day: 7–8.30 a.m., 10–12 a.m., 2–4 p.m., 5–7 p.m. and 8–9 p.m. on weekdays and 9–11 a.m., 12–2 p.m., 3–5 p.m., 6–7.30 p.m. and 8.30–10.00 p.m. during weekends. Each sampling point could be filled in for one hour, with the participants receiving a reminder prompt after 30 min, if needed. In total, there were 210 sampling points. Each sampling contained questions with predefined answering categories to enable rapid responses. Two questions asked where the participant was at that moment (categorized as being at home versus elsewhere) and with whom (categorized as being alone versus not alone). Then, questions asked about current emotions (‘At the moment, I feel…’), including nine different emotions each to be answered on a 7-point Likert scale (‘not at all’ to ‘very strongly’). The emotions were insecure, sad, angry, anxious, lonely, worried, ashamed, happy, and relaxed. These items were based on the PANAS scale [18] and in consultation with people with lived experiences. Finally, the participants were asked whether they had eaten much more than a regular meal or snack since the last assessment, using a 5-point Likert scale (‘not at all’ to ‘very much’). If answered with somewhat/quite a lot/very much, they received a follow-up question on whether they had experienced the feeling that it was hard to stop eating (LOC, Loss Of Control eating). Besides these fixed sampling points, the participants were able to report a binge eating episode if one occurred, using the same questions on overeating and LOC. The occurrence of overeating in combination with the LOC was defined as a binge eating episode. The reported emotions and contexts were tracked in the graph of the app using dots per registration; a binge eating episode was indicated with a vertical line in the graph (see Figure S1 for a fictitious example).

#### 2.3.3. Semi-Structured Interview

A semi-structured interview was held with the participants and practitioners to evaluate their experiences with the EMA application during BED treatment. The interview schedule was specifically designed for this study and covered the themes: burden of symptoms (participants only), experience of treatment as usual (practitioners only), impact of the EMA application on change in symptom burden and treatment course, and recommendations. The interview schedules for the participants and practitioners are presented in the Supplement (Figures S2 and S3).

### 2.4. Analyses

#### 2.4.1. Qualitative Analysis of the Interviews

Interviews were transcribed verbatim and analysed with codebook thematic analysis with theory-driven predefined main themes [19] in Atlas.ti 22 for Windows. This analysis was informed by Braun and Clarke’s canonical approach to thematic analysis and adopted a codebook-based analytic strategy that was considered appropriate for the relatively focused and technically oriented research question [20]. Codebook analysis was conducted as follows: first, during the open coding, relevant sentences from the transcripts were reduced to codes showing the sentence’s core. Next, axial coding was used to compare codes with each other. Similar codes were merged into thematically derived and theory-driven sub-themes. In the last step (selective coding), sub-themes were assigned to the broader predefined themes ‘feasibility’ (compliance, user-friendliness, burden, and readability and comprehensibility of the graph) and ‘usefulness of the app’ (new insights based on graph and reporting procedure, as well as contributions to reduce BED symptoms). We conducted open data-driven coding to ensure a data-driven and inclusive account of experiences. The fitting of the open/axial coding to theory-driven main themes ‘feasibility’ and ‘usefulness’ was performed at the end of the analyses (selective coding). The transcribing and coding were conducted by two junior research assistants, and theme formation was discussed in recurrent sessions with the juniors, the senior qualitative researcher and a senior binge eating researcher. Recruitment for interview participants was stopped when saturation was reached based on consensus among the researchers.

#### 2.4.2. Quantitative Analyses

We described sample characteristics and EMA responses using means (standard deviations, SDs) and percentages.

## 3. Results

### 3.1. Characteristics

Characteristics of the total sample (*n* = 20) and the sample for qualitative analyses (*n* = 16) are provided in Table 1. The two samples were quite comparable. The total sample (*n* = 20) consisted mostly of females (75%), with a mean age of 34.95 years (SD = 10.32) and a mean body mass index of 40.13 kg/m^2^ (SD = 8.11). On average, the participants scored lower on the EDE-Q post- than pre-treatment (2.56 versus 3.38). The mean EMA response rate for the total study sample was 31.1%, with nine participants having a response rate > 30% (>65 of 210 EMA reports were filled in; mean response rate of 56.4%). Compliance ranged from 0.9% to 84.3% among those who participated in the interviews (*n* = 16) and from 17.1% to 78.1% among those who did not participate in the interviews (*n* = 4), suggesting that the interviews were conducted with in a diverse group of participants. In total, only 20 binge eating episodes were reported in the app.

### 3.2. Results of the Qualitative Analysis

#### 3.2.1. Feasibility

Feasibility is defined as the merits and viability of the EMA app, which was covered in five themes during the interviews (see Table 2). Below, themes are denoted in italics, and subthemes in bold text. Quotes are denoted as Px for patients with anonymized participant code x, and as Ty for the practitioners (therapists and treatment coordinators).

#### 3.2.2. Compliance with EMA App

Compliance regarding the procedure was noted by the participants, indicating a few times that they used the app as much as possible. However, the participants more frequently noted that they often lacked the opportunity to fill in the app, e.g., because they were travelling (P12). Some participants mentioned that they stopped using the app early, e.g., ‘I think I really tried for 1.5 weeks’ (P5). Within the treatment groups, it was perceived as hindering that not all individuals used the app or quit prematurely, which affected the value of the app as part of group therapy. The practitioners noticed that group/social dynamics affected individual experiences, e.g., ‘in a negative group, more people tended to decline’ (T4), and that ‘some people joined out of loyalty with the therapist’ (T4). The practitioners also indicated that age might have affected participation, with the technology used being more appealing to younger participants. However, this was not reflected in the low response rates among the young adults (26 or younger), who indicated that they had difficulty incorporating time for registration into their daily schedules or who indicated that emotions were not relevant to their problems.

Compliance also regarded the content of the reporting. Several participants filled in emotions truthfully and without much thought, yet they differed substantially in registering binge eating. Some registered their binge eating episodes, while others did not for various reasons: they doubted whether the eating should be called a binge, did not want to report the binge, or were too preoccupied with the binge.

#### 3.2.3. User-Friendliness of the EMA App

Despite a high user-friendliness of the questionnaires (e.g., clear questions that can be answered quickly), compliance may have been jeopardized by practical issues and some technical problems. Practical issues prohibiting a response included not having the smartphone nearby the whole day, the short time window of questionnaire availability, and inconvenient timing of the questionnaires. The participants suggested improving the app by allowing them to set their own start and end time, and to broaden the time window for response. Technical issues regarded mostly the notifications, with some participants indicating that they did not receive all notifications. However, it could also be that the participants sometimes missed notifications. The practitioners echoed these comments, but ‘advised clients not to give up too quickly’ (T3). Note that over the course of the study, we changed the notification system, resulting in a steadier flow of notifications. The practitioners additionally noted that the app makes registration ‘easier’ and ‘better readable’.

The relevance of the questions also appeared as a user-friendliness subtheme. Some participants indicated that sufficient emotions were assessed, while others suggested including options to personalize emotions and circumstances. The latter was also noted by one participant who thought the app focused too much on emotions and should focus more on circumstances.

#### 3.2.4. Burden of the EMA App

The practical burden of the assessment raised contrasting experiences. Some participants experienced the app as positive and did not regard the reminders as distracting, while most participants noted that they got too many reminders, which were distracting for some. Also, most participants indicated that the assessment period was too long, with the app becoming annoying. The practitioners agreed with these comments.

The app also presented an emotional burden, as for some participants, the registration of feelings was confronting and evoked shame. Indeed, the practitioners warned that ‘the graph can cause a downward spiral if it does not go well’ (T5), although ‘it also has potential to increase awareness of emotions, which is often difficult for this patient population and thus can be a good thing’ (T5). Indeed, some participants indicated that the registration of feelings was fine because it resulted in self-insight and sometimes even brought relief, e.g., ‘it was sometimes nice to express that you aren’t feeling happy. And then afterwards it’ll be okay again’ (P9).

Unexpectedly, the app appeared to have an unintended negative side effect: the participants indicated it felt unpleasant or even like a failure when they missed an assessment round. This aligns with the practitioners’ thoughts that some participants ‘filled in the app out of perfectionism’ (T1), a personality trait that is linked with binge eating [21].

#### 3.2.5. Readability and Comprehensibility of the Graph

Generally, the participants were positive about the appearance and readability of the graph (e.g., ‘graphs were clear’ (P7), ‘because of the colours I thought it was clear’ (P8)). Further, they recognized the goal of the graph. Yet, both participants and practitioners indicated that reading and interpreting the graphical patterns of (many) different emotions over the course of multiple days or weeks was difficult. However, the practitioners emphasized that particularly ‘assessments over a longer trajectory would say more’ (T2).

Understanding the graph was a challenge for some, sometimes resulting in giving up (e.g., ‘So I noticed that I didn’t look at [it] anymore’ (P10)). For others, the pattern was understandable and recognizable, but they did appreciate help from the therapist to achieve this, which was confirmed by the practitioners. However, the practitioners also pointed at the limited time they had to discuss the app during the sessions. The participants recommended a better embedding of the app in the treatment sessions to allow for more discussion and explanation of the registration.

#### 3.2.6. Usefulness

Usefulness is defined as the EMA app being advantageous and helpful. Usefulness was covered by three themes in the interviews (see Table 2). Below, themes are denoted in italics, and subthemes in bold text.

#### 3.2.7. Gained New Insight Based on the Graph and Reporting

Most participants frequently indicated that the reporting and graph increased awareness of and insight into (the role of) their emotions. The participants said that ‘the graph helped to realize that I’m less happy than I thought I was’ (P4) and that ‘I noticed that [insecurity] came up often’ (P13). The participants’ responses also indicate that the daily reporting prompted reflection, e.g., a ‘check-in moment’ (P15), and ‘the app helped …, to think “what it is that you’re actually doing”?’ (P10). This is notable considering the low response of several participants: apparently, few registrations may already increase insight. Only a few participants reported that they did not gain new insights from reporting emotions or reading the graph, e.g., because they deemed the tracking of emotions as ‘a bit irrelevant’ as the participant described him/herself as ‘not an emotional person’ (P2). The practitioners added that the app may ‘induce a moment for reflection’ (T5) and ‘unravels subconscious emotions’ (T4), since it asks questions in ‘a real-life setting’ (T4) where the participants have to report in the moment instead of retrospectively.

#### 3.2.8. Whether the EMA App Helped to Reduce BED Symptoms

Only a few participants indicated that the app helped in reducing BED symptoms, with one participant stating that recognizing the role of emotions in the occurrence of binges helped him/her to prevent binge eating. However, others mostly attributed changes in symptoms to treatment rather than to the app. Despite this, the potential added value of the app was experienced positively by the participants and practitioners. Some suggested that the effect may have been larger if the app had been used later or after treatment, rather than at the start. The practitioners also recommended to ‘use the app as aftercare’ (T4), although they would ‘advise against using app as stand-alone treatment’ (T5).

## 4. Discussion

This uncontrolled implementation study evaluated user experiences with an EMA smartphone application as tool during CBT treatment for BED. The participants and practitioners indicated mixed experiences. Although the app was received as a user-friendly tool that increased reflection and insight into emotions for several participants, many also perceived the reporting procedure as burdensome because of the many registrations. Moreover, reporting emotions and binge eating episodes was considered confronting and shameful for some participants. Together, this may explain the low number of registrations and of reported binge eating episodes in the EMA application.

### 4.1. Interpretation of Findings

The low number of registered binge eating episodes contrasts with previous studies [9]. Nevertheless, it should be noted that our design differed substantially from most previous studies, which mainly conducted EMA assessments in non-treatment samples or prior to the start of treatment, while our EMA assessment took place during treatment. CBT treatment for BED often has a high initial treatment response, in which the number of binge episodes rapidly declines [22], which may explain the very low number of reported binge eating episodes in our sample. Furthermore, compliance rates in previous EMA research among individuals with an eating disorder generally exceed the 31% observed in our study. A recent review [9] indicated that of the studies using comparable signal-contingent reporting, 24 studies had compliance rates between 53 and 97%, while 12 did not report on compliance. Differences in design between our study and previous studies may explain the diverging numbers: nearly all studies were conducted pre-treatment, lasted for 7–14 days and offered substantial monetary rewards [9]. Perhaps a more comparable design was an EMI study in which participants were asked, among other tasks, to self-monitor their affect four times per day. The moderate initial compliance of 58% in the first two weeks deteriorated over the course of 19 weeks of treatment to 11% responding at least once a day in the last two weeks [11]. This may mirror a growing familiarity with emotions and contexts linked to binge eating that reduced the perceived need for continued monitoring. However, the participants indicated, just as in our study, that the daily registrations were too burdensome and repetitive [11]. Together, the decline in response rates observed across these studies suggests that it is worth evaluating whether a few weeks of reporting—rather than extended monitoring periods—are sufficient.

Along with the practical burden (e.g., a high number of registrations), our qualitative findings also suggest that the low compliance may be due to the emotional burden of the EMA app, such as the confronting element that several participants mentioned. Indeed, individuals with BED can experience difficulties identifying and describing their emotions (e.g., alexithymia) alongside difficulties in emotion regulation [3,4]. The participants indicated that the daily registrations helped them to reflect and increase their emotional awareness—similar findings were reported in recent qualitative EMA studies on self-harm [23] and eating disorders [11,24]. Considering the low number of registrations in our study, increased insight may occur with fewer registrations than initially expected, suggesting a relatively straightforward strategy to support emotional awareness. However, it is important to emphasize that the increased reflection and emotional awareness reported by some participants may also have been attributable to other components of CBT, rather than the daily app-based registrations. Future studies should ideally measure emotional awareness pre- and post-treatment and include both an intervention and a control group to be able to disentangle any potential effect of the EMA app.

In line with previous research [24], we noticed that some participants also described ‘feeling punished’ or ‘having failed’ after missing a registration, suggesting that the registration may activate pre-existing illness-related cognitions and emotions. Clinical perfectionism refers to a pattern in which an individual’s self-worth is heavily dependent on meeting highly demanding personal standards [25]. When these are not achieved, people tend to evaluate their performance in an all-or-nothing manner and engage in harsh self-criticism. Within eating disorders, clinical perfectionism is considered a transdiagnostic factor that contributes to the maintenance of symptoms [21]. Research suggests that perfectionistic concerns, including fear of making mistakes, doubts about one’s actions, and worries about negative evaluations, show a stronger association with binge eating behaviours than perfectionistic strivings [21]. The current study protocol, consisting of several prompts per day with a one-hour response window, may therefore have been perceived as another context in which performance was judged, instead of as a neutral monitoring procedure. Consequently, missing a prompt could be interpreted as evidence of personal inadequacy rather than as expected missing data, potentially eliciting self-criticism, guilt, and shame. This is clinically relevant, as shame has been implicated in a self-perpetuating cycle in which binge eating both results from and reinforces feelings of shame. Qualitative research on another self-monitoring app for eating disorder treatment similarly found that some patients felt judged or monitored, omitted shameful symptoms, or engaged in obsessive logging [26]. This risk is not unique to digital monitoring, as conventional self-monitoring is also often approached perfectionistically. Therefore, measurement-related loyalty may serve as an indicator of perfectionism, enabling therapists to identify and address these tendencies during treatment.

### 4.2. Strengths and Limitations

The strength of this study was the in-depth qualitative overview of the applicability of a smartphone application during treatment, which highlighted the contrasting experiences between users. However, the study’s findings should also be interpreted considering some limitations. The first limitation was that although the designed application was tested many times before the start of the study, notifications were not always received by all participants in the first two treatment groups due to technical issues. These issues influenced compliance rates and reduced generalizability. Indeed, the mean response rate in the first two treatment groups was 21.0%, and after changing the method of notifications for the last three treatment groups, the mean response rate was 33.8%. However, it is not possible to determine to what extent this difference was due to technical problems or whether the app became more embedded in treatment. Secondly, our findings might also be influenced by the underreporting of binge eating in the EMA app. Although this could reflect an initial treatment response, it may also be the result of socially desirable reporting, as some participants indicated in the interview that they did not report all binge eating episodes. The low number of reported binge eating episodes limited quantitative estimations of the relationship between affect and binge episodes, and while broader overeating behaviours reflect more variance, these capture only certain aspects of binge eating. Thirdly, over the course of the study, there was a high turnover of therapists because of the aftermath of COVID-19, illness, or transfers. This might have influenced the engagement of therapists in discussing the app during sessions, but at the same time, it also reflects commonly occurring treatment circumstances. Finally, by involving researchers with complementary clinical and theoretical expertise, we aimed to develop a balanced and nuanced interpretation of the data. Nevertheless, we acknowledge that the research team was not fully representative in terms of broader sociodemographic characteristics, which may have influenced data interpretation.

### 4.3. Recommendations

The added value of this study lies in the qualitative evaluation of the implementation of an EMA smartphone application during BED treatment. Overall, the app was considered a user-friendly tool by most participants, while several participants also indicated that the registrations were helpful in increasing awareness of (fluctuations in) emotions. This pattern was also observed in other qualitative EMA studies among BED patients [11,24], as well as in other clinical samples [23] and among adolescents in the general population [27], highlighting the broader potential of EMA to enhance awareness and serve as a valuable tool both within and beyond the clinical treatment context. Drawing on our real-world example of implementing an EMA application and the cautiously positive feedback from some participants and therapists, we outline several recommendations to enhance the feasibility of EMA during treatment for BED, as well as potentially for other psychiatric conditions.

Firstly, the implementation may benefit from a shorter assessment period and better alignment with the content and course of treatment. For example, a one-week assessment at the start of treatment may help patients focus on real-time emotional experiences, whereas a one-week assessment later during treatment aligns with the timing of the emotion-regulation module of CBT, which targets the connection between emotions and binge eating symptoms [28]. Like previous research [11,23,24], the participants indicated that the reporting enhanced reflection and insight in their emotions, which apparently, considering the general low response of the participants, may already be reached with a few registrations. So, in contrast to EMA research, weeks-long daily registrations and strict compliance may be less important for treatment purposes and should be avoided to minimize potential negative cognitions and emotions associated with missed registrations.

Secondly, in line with the previous suggestion, EMA should be presented as a neutral self-observation tool rather than an assessment of adherence, with missed prompts explicitly framed as acceptable. Clinicians should regularly explore patients’ attitudes towards both completing and missing registrations and should consider modifying or terminating monitoring when it appears to intensify self-criticism, shame, or eating disorder symptoms. Because the present study did not investigate symptom deterioration caused by the app itself, the findings indicate a plausible risk mechanism rather than proof of clinical worsening.

Thirdly, the EMA application should be well-embedded in treatment. To effectively and meaningfully incorporate EMA or any other tool into clinical practice (research), it is recommended to offer therapists the relevant comprehensive knowledge of the tool’s theoretical foundations and scientific evidence regarding therapeutic advantages. While scientific evidence is still developing, real-time monitoring of emotions, contexts and behaviours aligns well with the transdiagnostic model of eating disorders [28] and can be meaningfully integrated within this framework by making use of modern technology. In addition, therapists should have sufficient allocated time to discuss registrations during treatment sessions. Such prerequisites will help to avoid uneven utilization of treatment tools by practitioners.

Fourthly, given the rapid advancements in digital health technologies over recent years, it is further recommended that clinicians and researchers leverage existing m-health platforms, such as Avicenna and M-path, to implement daily self-monitoring with greater ease and efficiency in their target groups. These platforms were still in their infancy when we started developing our application but now provide sound digital infrastructures that enhance the feasibility, scalability, and ecological validity of EMA protocols, while also facilitating data management and integration into therapeutic workflows. Indeed, clinician dashboards allow therapists to easily create and adjust EMA schedules, including the ability to tailor items to individual patients—an important feature considering evidence that the emotions that are most relevant for binge eating vary from person to person [29]. Although simply registering emotions and eating patterns already appeared to heighten the participants’ awareness, graphical data visualizations may offer additional support for some individuals, and the dashboards of m-health platforms make it possible to visually review emerging patterns during sessions. Existing m-health platforms are widely applicable across all major smartphone operating systems and are continuously updated, ensuring stable performance and alignment with evolving technological and clinical needs. As such, the use of existing m-health platforms will likely be less costly, testing will take less time, and technical issues are likely to remain limited, resulting in smoother and more effective implementation.

Finally, as not all participants were keen on using the smartphone app, we recommend offering the EMA application as an add-on tool rather than incorporating it as an integral part of BED treatment. Potentially, personalized content in the EMA application, such as choosing relevant emotions and situations, may increase compliance. Given that this study is one of the first to examine the feasibility of an EMA application during BED treatment, more research is needed to replicate and extend our findings while incorporating these recommendations. It would be particularly helpful to evaluate the effectiveness of adding EMA or EMIs into CBT, as well as their potential for preventive efforts and for bridging the waiting-list period before treatment, using a rigorous design (e.g., randomized controlled trial).

## Figures and Tables

**Table 1:** Descriptive characteristics of the study sample.

Sample Descriptives		*N* (%) or Mean ± SD	
		Total (*n* = 20)	Sample Qualitative Analyses (*n* = 16)
Biological sex	Female	15 (75.0)	12 (75.0)
	Male	4 (25.0)	4 (25.0)
Age (years)		34.95 ± 10.32	33.44 ± 9.92
Educational level ^1^	Low	1 (5.0)	0 (0.0)
	Medium	11 (55.0)	10 (62.5)
	High	8 (40.0)	6 (37.5)
BMI (kg/m^2^)		40.13 ± 8.11	39.56 ± 8.89
EDE-Q baseline score ^2^		3.38 ± 1.28	3.73 ± 1.19
EDE-Q post treatment score ^3^		2.56 ± 1.64	2.60 ± 1.70

Notes: ^1^ High = higher vocational education or university; medium = higher secondary school and lower vocational training; low = lower secondary school or less. ^2^ *N* with missing data: *n* = 6 in total sample, *n* = 2 in quantitative sample, *n* = 5 in qualitative sample. ^3^ *N* with missing data: *n* = 6 in total sample, *n* = 2 in quantitative sample, *n* = 6 in qualitative sample.

**Table 2:** Thematic analysis of the interviews including example quotes.

Theme	Sub-Theme	N ^#^	Example	Participant
FEASIBILITY Compliance with EMA				
	Could not always fill in the app	10	“Quite quickly after you receive a reminder you should fill in the app. I failed to do that 5 times a day. Often I was on the road or it came at a time when it really didn’t work out.”	12
	Used app as much as possible	3	“It took some time getting used to that it could be random. […] if I did see it [the reminder] popped up, then I tried to make time for it or I just went to the toilet to fill that in.”	15
	Answered without much thought	10	“If you read the same sentences every time, you quickly don’t use much thought.”	9
	Non-compliance of group	6	“I was the only one [who used the app].”	13
	Stopped using the app soon	8	“I think I really tried for 1½ week. In the end, it was on [my phone] for 3 weeks.”	5
	Did not look at the graph	2	“I have to admit, I think I didn’t really look into the results [from the graph].”	11
	Registering binge eating			
	Yes	3	“In the end, I had binges and I filled those in the moment I realized.”	14
	Sometimes	3	“Not always [used the app during a binge], but occasionally.”	2
	No	5	“Three [binges], but I didn’t fill those in. [….] clearly you’re busy with completely different things at that moment. You’re only focused on that binge.”	7
			“I don’t know how quickly I would [fill in a binge]. Because it’s only since last week that I contact somebody when I tend to binge. I would always really keep that to myself.”	9
	Filled in app truthfully	5	“I had more difficulty with [honestly answering eating] registrations than with the app.”	1
	Access of others			
	Would make a difference	6	“I think that [if others had access] I would have been even less honest, because then you get the idea that other people will have an opinion about it as well.”	5
	No difference	9	“Well, [honestly filling in] depends entirely on the group. These are all nice people so in my case, it would not have made a difference.”	2
User-friendliness of EMA				
	Appearance of the app			
	Positive	1	“I actually thought it looked nice, well-organized, easy, not too much fuss, not too many detours, that was well thought-out.”	10
	Negative	2	“It looks a bit boring. […] Maybe something with a smiley or something that pops up when you’ve completed it. Like ‘well done’ or ‘keep going’.”	9
	Organization of the app	11	“The look [of the app] is clear, because is it very structured.”	11
	Questions were clear	12	“Questions were fine and clear and also clearly arranged.”	10
	Answering is easy and quick	2	“I also found it very easy to fill in […] within 2 min you were done.”	7
	Vary questions	3	“If you read the same sentences every time, you quickly don’t use much thought. Whereas if you’d ask in just a different way you think ‘I hadn’t thought of it that way’.”	9
	Assessment of emotions			
	Sufficient	2	“I thought those [assessed emotions] were sufficient.”	2
	Option to add emotions	2	“I missed that. You just had a list of emotions you could choose from, but the emotions you were experiencing at that moment were not there.”	11
	Too focused on emotions	1	“Maybe [better if] questions focussed less on emotions and more on circumstances.”	3
	Missed certain answer options	2	“There was a question whether you ate a larger portion than normal, the last question, and I missed some kind of neutral answer option there.”	1
	Option to add notes	8	“Perhaps a space to fill in something yourself. […] at some moments you can say I feel like this right know. So apart from the standard questions that already exist.”	13
	Time slot to fill in app too short	6	“It felt like I didn’t really get the chance to always fill those in. Because I’d be too late or it wasn’t possible anymore.”	3
	Phone not always close by	3	“Nobody uses their phone the entire day. Not even in my spare time.”	1
	Setting own start & end time	3	“Or that you could set your own timeframes. […] that I can choose my last will be 9 o’clock, the time I sometimes go to bed.”	4
	All registrations in one app	7	“That you make a combination with registering your food. Because now we used two apps that are partly overlapping.”	8
	Problems with notifications	13	“Then there were weeks where I didn’t get a notification at all. It didn’t occur to me [the notifications stopped] until I heard it from others from the therapy group.”	1
	Technical issues app	7	“At a certain point, I stopped using the app because I had to log in every time.”	9
Burden of the measurement				
	Experienced the app well	5	“Overall, I liked [the app] a lot.”	2
	Assessment period too long	12	“6 weeks is really long to receive the same questions every day. […] At a certain point it became irritating, but it certainly did trigger something in the beginning.”	10
	Too many reminders	10	“You can’t fill in [the app] 6 times a day. […] It just didn’t fit my schedule.”	3
	Were reminders distracting?			
	Yes	3	“When it notified you, I’d really lose focus if I was studying or at work.”	5
	No	7	“Because you were quickly done with the questions, I didn’t mind. It was not distracting.”	1
	Too much work	4	“It was a lot. You start with therapy and get an app where you only have 1 h to respond.”	4
	Consequence of registering			
	Felt ashamed	2	“You don’t feel good because you’ve had a binge, you feel guilty and you’re ashamed. That does come up again [when filing in the app].”	6
	Confronting	6	“[Filling in] was very confronting. You do have to be able to handle that as a person.”	2
			“[Filling in emotions] was okay because it made you more conscious. But I also remember, sometimes it was really like you think there we go again. Or that you would skip it because you didn’t feel like checking in. Yes, but no 8 out of 10 times I did manage to go through that [feeling]. But you’d feel a lot of resistance sometimes.”	15
	Relief	2	“It might sound weird, but sometimes it’s nice to express that you aren’t feeling happy. And then afterwards it’ll be okay again.”	9
	Self-monitoring			
	Difficult	5	“I find it difficult to name that for myself. Because I’ve lived with it for so long that it doesn’t always feel like a problem but more like who I am.”	13
			“It’s just difficult sometimes […] because […] how insecure do you feel? I almost always feel insecure. Then I look at this moment, is it different now?”	1
	Fine	4	“I really liked [the self-monitoring], as it makes you realize how you are put together.”	4
	Unpleasant to miss registration moments	5	“If you’re at work or busy with something you don’t look at your phone all the time. […] on the one hand I get it, it [concerns] how you are at that moment. But I also found it a bit unpleasant, because I missed it a few times.”	13
			“It felt like I was being punished a bit because I couldn’t fill it in anymore after an hour. So I’d think: ‘too late again’ […] That I would feel worse of it.”	5
			“It felt a bit like a failure or something if I couldn’t [fill it in] once. I discussed that and [the therapist] said I should make that choice for myself. If it wouldn’t help me, I’d better stop.”	4
Readability of the graph				
	Graphs were clear	8	“The lines [from the graph] were clear and I could see how it was meant.”	7
			“Because of the colours I thought [the graph] was clear.”	8
	Graphs were nice	2	“I actually really liked that [the graph]. I’ve nothing to comment on.”	5
	Graphs were too dense			
	Lines (emotions) too close	10	“If I looked at the overview, then I saw a very small screen with lots of lines, nothing else.”	2
	Multiple days is not clear	4	“For a day, I see the overview. But to see how the last 8 weeks were, I’d be busy for a while.”	2
	Bar chart perhaps clearer	2	“I think that the lines make it a bit messy. If you’d colour it with bars, it would be clearer.”	11
Comprehensibility of the graph				
	Recognized patterns in graph			
	Yes	9	“I knew that for me it was sadness and stress, and I could see that [in the graph].”	6
	No	2	“But of course, [reading the graph] is more difficult with that many measurement.”	10
	Understanding the graph			
	Could not draw conclusions	2	“But I couldn’t get that from [the graph]. […] I couldn’t draw conclusions from that.”	2
	Did not understand graph	5	“I noticed that I didn’t look at [the graph] anymore. Whatever, I only see a jumble of lines intersecting from different points.”	10
	Needed more explanation	3	“Or maybe more explanation about it, how we should look at the graph.”	7
	Discussed graph with therapist			
	Yes	2	“[The practitioner] showed you how things were during the session. […] They do see something in the graph which I didn’t really. That’s just a piece of experience you miss.”	6
	No	10	“No, there wasn’t really feedback on that, because not everyone participated.”	10
	Propose more discussion	14	“If [the app] was more part of treatment and the therapists would discuss it, I think it [insight] could be more.”	13
USEFULLNESS				
Gained new insight based on the graph				
	New insights in emotions			
	Yes	19	“The app [graph] mostly helped me realize that I’m less happy than I thought I was.”	4
			“I noticed that the [emotions] I told you about, that I saw those back in the graphs. And that was more of an indication of ‘oh yes’. So I think insecurity was one, […] and I noticed that that came up often. That often fluctuated.”	13
	No	3	“I was curious about what the app [graph] would teach me about myself and at what moments I get into a binge. I couldn’t draw any clear conclusions from that.”	12
Gained new insight based on registration				
	Tracking emotions is unnecessary	2	“I’m not a very emotional person, so questions about emotions were a bit irrelevant for me.”	2
	Increased awareness of emotions	25	“It [the app] just made it a check-in moment. So what do I feel now? What am I actually doing? […] That helped me like ‘oh, I feel tense now for I’m going through this’.”	15
			“[It] made me more aware of emotions that played a role, as I often don’t think about that.”	13
			“On the day of treatment, so when you have the group, you are very busy with [your feelings]. Then you cycle home, step into you normal life and sometimes tend to forget [the treatment]. The app helped with that, to think ‘what it is that you’re actually doing’?”	10
Whether app helped to reduce BED symptoms				
	Symptoms changed due to app:			
			“And if you feel the urge to eat, where does it come from? […] now it’s because I’m tired, now it’s because I’m tense, because my brother is sick, […]. If you know where it comes from, then you can think what would help now?”	15
	Yes	2	“If you have multiple [registrations] per day and you later look back at those. I had a bad day today, so it’s actually quite logical that the eating disorder is active. That’s something I have to keep an eye on, that I eat breakfast earlier in the morning or that I prepare myself for an unpleasant conversation. You do get insight because of [the registrations].”	9
	No	6	“At the moment, it’s more the rest of the treatment [that helped].”	8
	Both app and treatment	2	“I think both.”	16
	Potential added value			
	Yes	10	“Yes, I think it could be an added value at the right time.”	16
	No	6	“Because it was already clear for me what my bottlenecks are in that area.”	2
			“I do think that the eating diaries that we use are so extensive that your app is more supportive than an addition really.”	12
	Would recommend app			
	Yes	6	“I can imagine that such an app can really work in the treatment process. I do think that there are still some points of improvement. But that is why we [tested] it.”	10
	No	5	“That’s really personal […] for me it’s just not of added value.”	7
	Apply app later in treatment	6	“I do believe that the app can do something but I’m inclined to say that it can do more in a later stage of treatment than in the beginning.”	10

^#^ Indicates the number of times that this subtheme was mentioned by different participants and within interviews (counted twice for a participant if this subtheme was mentioned twice at separate moments during the interview).

## Data Availability

The data presented in this study are available on request from the corresponding author due to privacy and ethical restrictions.

## Supplementary Materials

The following supporting information can be downloaded at https://www.mdpi.com/article/10.3390/nu18183046/s1: Figure S1: A fictitious personal graph from the EMA smartphone application, as an example; Figure S2: Interview Schedule Participants (translated from Dutch); Figure S3: Interview Schedule Practitioners (translated from Dutch).
