# Supplementary material for: Qualitative Feasibility and Usability Study of an Ecological Momentary Assessment (EMA) Smartphone Application in Binge Eating Disorder Treatment
Source: Nutrients. 2026 Sep 17;18(18):3046. doi: 10.3390/nu18183046 (PMC13610177; doi:10.3390/nu18183046)
Supplement: Supplementary file 1 [file nutrients-18-03046-s001.zip › nutrients-4437676 supplementary.pdf]

Supplementary Materials

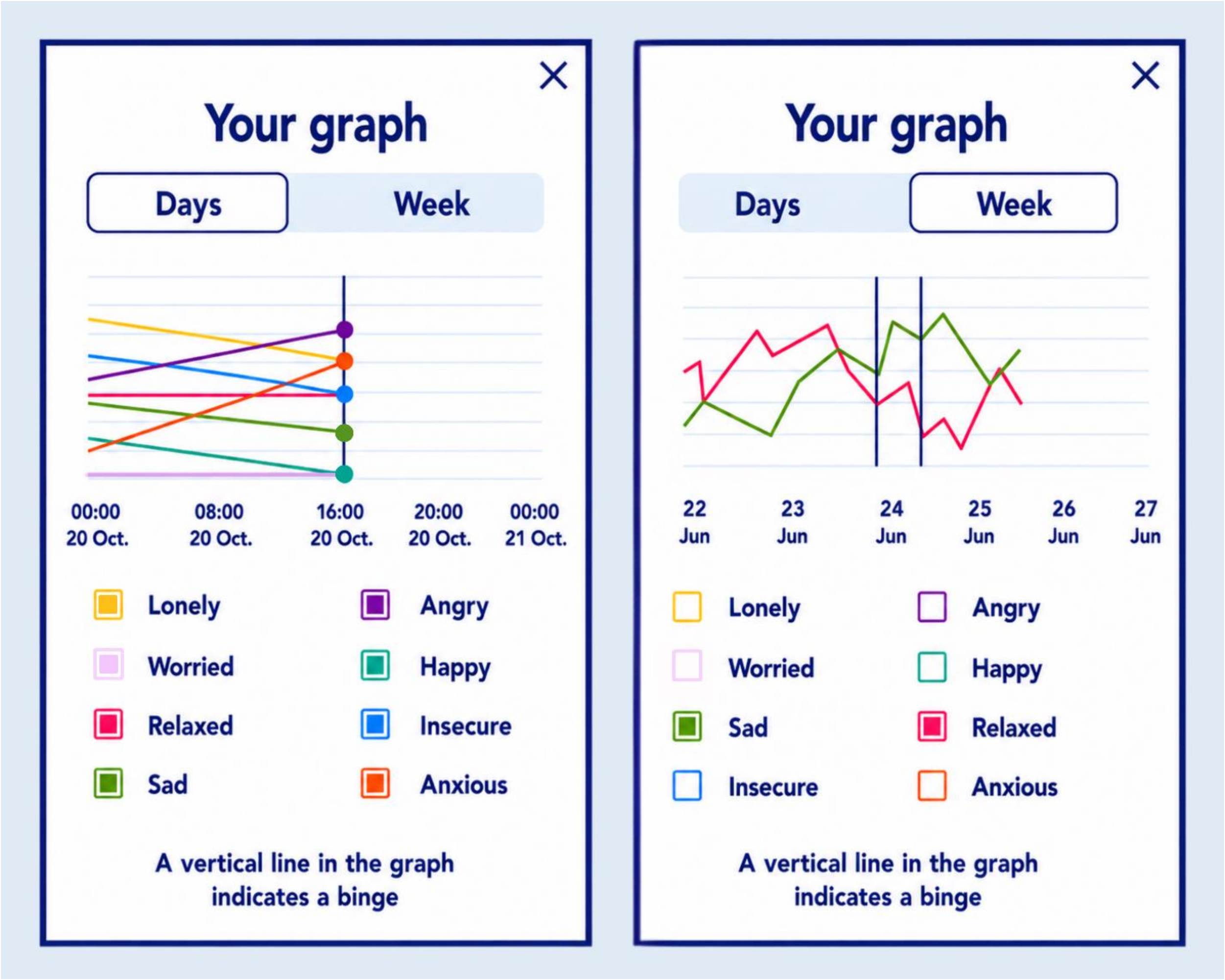

Figure S1. A fictitious personal graph from the EMA smartphone application, as an example

Figure S2. Interview Schedule Participants (translated from Dutch)

Introduction (5 min)

Welcome & thank you for your participation

During this interview we would like to hear about your experience with the new app that was used during the treatment for binge eating. We would like to know more about how you used the app, what you thought of it, and what the app has meant to you.

We would like to ask you to tell us about your experiences in your own words. It doesn't have to be a beautifully detailed story, you can just tell what comes to mind. It doesn't matter whether those are positive or negative experiences! There are no right and wrong answers, and the interviewer will not be surprised by any answers.

The interviewer will ask questions and may ask for explanations and details. If you do not understand a question or find it difficult, you can ask the interviewer for clarification. If you don't want to or can't answer a question, that's always okay. If you want to take a break or finish the interview, you can always indicate this.

The interview lasts approximately 45 minutes. Do you have any questions before we start?

Do you agree to this interview being audio recorded?
I will start recording now.

A. Spontaneous reporting and prioritizing (5 min)

Let's start with your user experiences.

a. Can you tell us how you experienced the app?

Check for the level of concrete use:

- User-friendly
- Frequency
- Questions

Points of interest in interview intro:

- Invite spontaneous speaking
- Ask for prominence of experiences
- General tenor: positive, negative, double, ambivalent?

B. Experience/Insight in complaints (10 min)

You told me about your experience with the app itself. To get a good idea of the role that the app has played in the treatment of your complaints, I would like to ask you a number of questions about your complaints.

b. *Would you mind telling us with which complaints you started treatment?*

Check for:

- Symptom level (listen/ask beyond psychiatric jargon to indicate the level of experienced complaints)
- Experience level: *What was that like for you?*
- Functional level: *How much trouble did you have/how were you hindered by these complaints in your daily life?*
- Evaluative/insight: *Looking back, which complaints were most prominent for you?*

c. *Can you tell me how you are doing now?*

- *Do you still suffer from the complaints you just described?*
- *What changes have you noticed in yourself?*
- *Have other issues come to the surface that you previously did not experience or experienced less?*

C. Impact app on changes in complaints (5 min)

d. *You have described some changes. Can you attribute these changes to the app and/or other treatment components?*

Check for:

- Relationship between app and other treatment components
- Impact treatment compared to doing nothing

e. The app asks you to keep an eye on your own complaints. This is a kind of 'self-monitoring'. How did you experience this?

Check specifically for:

- Patterns in complaint experience/course
- Triggers individual/contextual

f. You told me about changes you experience in your complaints (b) and you indicate that these changes were initiated by using the app (e). Have these changes caused you to interpret certain questions in the app/questionnaires differently?

D. Impact app on course of treatment (10 min)

To get a good sense of the role that the app has played in your treatment process, I would like to ask you a number of questions about how you experienced your treatment process.

g. Can you outline what your treatment process looked like?

Check for:

- o Type of treatment
- o Group/Individual Ratio

h. How did you generally experience this treatment process?

[=Control question] check impact of general negative/positive attitude about treatment on app experience

i. You shared the information you collected in the app with your practitioner(s). How did you experience this?

- o *What role did this data collection play in your treatment?*
- o *Would your experience with the app have been different if your practitioner did not have access to your answers?*

j. You also shared the information collected in the app with your group in group therapies. How did you experience this?

- o *Do you think it would have made a difference whether you were in a group or individual therapy program?*

k. Looking back on your own treatment process: Do you see the app as added value to the treatment process?

- o *Why/why not?*
- o *Under what conditions?*

E. Should we continue this? (5 min)

l. Based on your experiences: would you recommend using the app in the treatment process for binge eating?

- o *What would you advice the researchers/developers?*
- o *What would you advise users (practitioners & patients)?*

m. Are there things you missed in the app?

- o *Are there any disadvantages/pitfalls that researchers/developers should know about?*
- o *Are there any disadvantages/pitfalls that users (practitioners & patients) should know about?*

F. Final remarks (5 min)

m. Is there anything else that I have not asked, but will be important to have a full understanding of your story?

n. How did you experience this interview?

Thank you very much for your participation.

As agreed, you will receive an information letter with our research findings once this study has been completed.

If you have any questions after this interview, or would like to add something, you can always send the researchers a message.

If you notice that this interview raises a new healthcare need for you, please let the researchers know. We are always happy to brainstorm with you.

Thank you again for your participation.

Figure S3. Interview Schedule Practitioners (translated from Dutch)

Introduction 5 min
Welcome & thank you for your participation

During this interview we would like to hear about your experience with the new app that was used during the treatment for binge eating. We would like to know more about how you used the app as a practitioner, what you thought of it, and what the app has meant to you.

We would like to ask you to tell us about your experiences in your own words. It doesn't have to be a beautifully detailed story, you can just tell what comes to mind. It doesn't matter whether those are positive or negative experiences! There are no right and wrong answers and the interviewer will not be surprised by any answers you will give.

The interviewer will ask questions and may ask for explanations and details. If you do not understand a question or find it difficult, you can ask the interviewer for clarification. If you don't want to or can't answer a question, that's always okay. If you want to take a break or finish the interview, you can always indicate this.

The interview lasts approximately 45 minutes. Do you have any questions before we start?

Do you agree to this interview being audio recorded?
I will start recording now.

A. Spontaneous reporting & prioritizing (5 min)

Let's start with your user experiences.

a. Can you tell us how you experienced the app?

Check for the level of concrete use:

- User-friendly
- Frequency
- Questions

Points of interest in interview intro:

- Invite spontaneous speaking
- Ask for prominence of experiences
- General tenor: positive, negative, double, ambivalent?

B. Experience with treatment as usual (10 min)

You told me about your experience using the app during the treatment. To get a good understanding of the role that the app has played during treatment of your clients, I would like to ask you a number of questions about how group treatment and the treatment process looks like.

b. *Would you like to briefly explain treatment as usual and what the process looks like?*

Check for indication of:

- Structure and content (ask what is offered during a regular treatment session)
- Experience level: *How do you experience providing group treatment? (what is going well, what could be better?)*
- Functional level: *how many symptoms did your clients have/how were your clients hindered by these symptoms in their daily life? To what extent has this decreased because of treatment?*
- Evaluative/insight: *To what extent did the clients' complaints decrease during their treatment? What has changed for your clients?*

C. Impact app on change in complaints (5 min)

c. *You described a number of changes among your clients during treatment. Can you attribute these changes to the app and/or other treatment components?*

Check for:

- Relationship between the app and other treatment components
- Impact treatment compared to doing nothing

d. *With the app, clients were asked to keep an eye on their own complaints. This is a kind of 'self-monitoring'. How did you experience this as a practitioner?*

Check specifically for:

- Patterns in experience/course of complaints
- Individual/contextual triggers

e. *Have you, as a practitioner, started to interpret your clients' complaints differently due to the overview of the questions in the graph? Has this overview led to new insights into complaint patterns?*

f. *Has the pattern of complaints resulted in new insights among clients?*

D. Impact app on course of treatment (10 min)

To fully understand the role the app has played in the treatment process, I would like to ask you a number of questions about how you experienced the treatment process.

- *g.* *Clients shared the information that was collected with the app with you as a practitioner. How did you experience this?*
  - *What role did data collection play within treatment?*
  - *Did the information provide (new) starting points for treatment?*
  - *How did you experience the practical use of the app?*

- *h.* *Looking back on the treatment process: Do you consider the app to be of added value to the treatment process?*
  - *Why/why not?*
  - *Under which conditions?*

**E. Should we move forward with this approach? (5min)**

- *i.* *Based on your experiences: would you recommend using the app during treatment for binge eating?*
  - *What would you advice the researchers/developers?*
  - *What would you advise users (practitioners & patients)?*

- *j.* *Are there things you missed in the app?*
  - *Are there any disadvantages/pitfalls that researchers/developers should know about?*
  - *Are there any disadvantages/pitfalls that users (practitioners & patients) should know about?*

**F. Final remarks (5 min)**

- *k.* *Is there anything else that I have not asked, but will be important to have a full understanding of your story?*

- *l.* *How did you experience this interview?*

Thank you very much for your participation.

As agreed, you will receive an information letter with our research findings once this study has been completed.

If you have any questions after this interview, or would like to add something, you can always send the researchers a message.

Thank you again for your participation.
